# Comment on Băetu et al. Beyond Trauma-Induced Coagulopathy: Detection of Auto-Heparinization as a Marker of Endotheliopathy Using Rotational Thromboelastometry. *J. Clin. Med.* 2024, *13*, 4219

**DOI:** 10.3390/jcm14041037

**Published:** 2025-02-07

**Authors:** Herbert Schöchl, Nikolaus Hofmann, Johannes Zipperle

**Affiliations:** 1 Ludwig Boltzmann Institute for Traumatology, The Research Centre in Cooperation with AUVA, Department of Translational Anesthesiology and Pain Medicine, 1200 Vienna, Austria; johannes.zipperle@trauma.lbg.ac.at; 2 Paracelsus Medical University, 5020 Salzburg, Austria; 3 Department of Anesthesia, Intensive Care Medicine and Pain Medicine, Medical University of Vienna, 1090 Vienna, Austria; nikolaus.hofmann@meduniwien.ac.at

With great interest, we read the recent study by Baetu et al. reporting “auto-heparinization” in a cohort of major trauma patients published in *J. Clin. Med.* 2024, *13*, 4219 [1].

The inner layer of the endothelium, the glycocalyx, contains negatively charged, antiadhesive and anticoagulant substances [2,3]. Severe shock and hypoperfusion result in glycocalyx damage and the release of “heparin-like” components such as heparan sulfate, dermatan sulfate or chondroitin sulfate into the blood stream. This mechanism has been incorrectly termed “auto-heparinization” [4]. 

Since the first description of “auto-heparinization” in only 4 out of a cohort of 77 trauma patients by Ostrowski et al., this phenomenon has gained increasing interest as a potential contributor to trauma-induced coagulopathy and a possible target in trauma bleeding management [5]. Despite it being considered a potential driver of trauma-induced coagulopathy, scientific evidence regarding both the incidence and clinical consequences of auto-heparinization is still poorly defined [6,7]. Therefore, further scientific work on this phenomenon is highly appreciated.

The diagnosis of “auto-heparinization” is still challenging. One possible option is the use of visco-elastic testing. A comparison of an intrinsically activated, heparin-sensitive test such as the INTEM clotting time (ROTEM/ClotPro) or CK r-time (TEG) applying contact activation by ellagic acid or kaolin and simultaneous measurement with an intrinsically activated test containing heparinase (HEPTEM, ROTEM/ClotPro or TEG CKH-test) could theoretically solve this diagnostic challenge. If the blood sample contains heparin, these heparinase-based assays inactivate the remaining heparin, and, subsequently, the HEPTEM-CT (ROTEM/ClotPro) or CKH r-time (TEG) should be shorter than the corresponding INTEM-CT or CK r-time [8].

Baetu et al. provided data on 217 severely injured trauma patients, of whom, according to their definitions, 12.9% displayed signs of “auto-heparinization” [1]. After carefully reading the manuscript, we would like to express some concerns regarding the interpretation of the provided data.

First, the most important parameters of the study, namely, INTEM-CT and HEPTEM-CT values, are missing. Furthermore, it is completely unclear at which time point ROTEM analyses were performed. These are essential parameters for correctly interpreting the data. Moreover, patients’ characteristics upon admission are missing, and no baseline data were provided on patients with and without “auto-heparinization”. Were there any substantial differences in blood pressure, heart rate, ISS, injury patterns or laboratory values? Interestingly, the authors provided a variety of figures depicting data upon admission and after 6 h without any context related to the focus of the study, namely, “auto-heparinization”.

Second, the authors defined “auto-heparinization” in trauma as a ratio of INTEM-CT/HEPTEM-CT of >1.25 and referred to the review article by Görlinger et al. [9]. Interestingly, Görlinger and colleagues recommend in their trauma bleeding management algorithm that if the INTEM-CT/HEPTEM-CT ratio is ≥1.25, protamine (0.3–0.5 mg/kg BW) should be considered. However, this recommendation is made without any evidence as no study has yet investigated such a treatment strategy. Moreover, Görlinger et al. referred, in this review, to the Ostrowski et al. study, where such intervention has been neither recommended nor performed [5]. To the best of our knowledge, there is no study that conclusively demonstrated that this arbitrary ratio is valid for trauma patients. Unfortunately, the authors did not provide any clinical data supporting that “auto-heparinization” has a clinical impact on bleeding tendencies. The authors could not demonstrate any significant differences regarding red blood cell transfusion between patients with an INTEM-CT/HEPTEM-CT ratio ≥1.25 compared to the “non-auto-heparinization group”. However, FFP transfusion rates were significantly higher in the “auto-heparinization” group compared to patients with “auto-heparinization” [2 (0–3) vs. 1 (0–3)]. However, from a clinical point of view, it is unclear why these patients received plasma transfusion at all.

Third, it is highly questionable whether heparan sulfate, which is released into the blood stream in the course of glycocalyx shedding, impacts INTEM-CT. Our group could not demonstrate any difference between INTEM-CT and HEPTEM-CT in a large number of trauma patients. In particular, those with severe hemorrhagic shock (base deficit >10 mmol/L), who carry the highest risk of glycocalyx damage and the release of “heparin-like” substances into the blood stream, demonstrated no differences between INTEM-CT and HEPTEM-CT [10]. Hemorrhagic traumatic shock in rats resulted in a threefold increase in heparan sulfate but did not result in any difference between INTEM-CT and HEPTEM-CT. Moreover, we spiked blood samples with heparan sulfate up to amounts which were almost two times higher than the highest previously reported value in trauma patients, with absolutely no impact on INTEM-CT. In contrast, heparin elicited a significant prolongation of INTEM-CT even in low amounts. Thus, heparan sulfate does not impact INTEM-CT, and the diagnosis of “auto-heparinization” is simply not possible with the available intrinsically activated assays [10].

Fourth, the exclusion criteria provided by the authors are hard to understand. Calcium levels outside normal ranges (not exactly defined in the manuscript), initial resuscitation following a massive transfusion protocol and patients who underwent emergency surgical procedures such as damage control surgery were excluded. Exactly these patients are prone to massive transfusion and susceptible to glycocalyx damage and the assumed “auto-heparinization”. So what is the rational for excluding these most severely injured patients from further analysis?

Fifth, it is unclear why only 42 patients demonstrated a clinical suspicion of “auto-heparinization”, in particular, as the median baseline values of base excess with −10 mmol/L (−15.8–−4.4) confirm severe shock. Interestingly, the authors reported an association between lactate levels and noradrenaline infusion and “auto-heparinization” but not for base excess. This is of particular interest as the median lactate levels were only 2.4 (1.1–4.2) in the “auto-heparinization” group vs. 1.8 (0.78–2.9) in the non-auto-heparinization cohort, therefore lower than the base excess values.

Sixth, the authors did not clarify what they meant by a “clinical and paraclinical suspicion of auto-heparinization”. It is also very hard to understand that a clinical suspicion for “auto-heparinization” was defined as a decrease in hemoglobin of >0.5 g/dL at 24 h. It is a very common finding in severely injured patients that hemoglobin levels decrease due to clear fluid administration, particularly those with a strong inflammatory response syndrome.

Despite the fact that this phenomenon is termed “auto-heparinization” by the authors, heparin is not released into the fluid phase in trauma patients with major shock. Thus, the precise wording for this phenomenon is not “auto-heparinization” but “auto-heparanization” [10]. The clinical impact of auto-heparanization is still uncertain. Unfortunately, the provided manuscript did not shed further light on this special trauma-related pathophysiology. It would be highly interesting to see the raw data of these patients.
